# A Worsening Cough: An Unusual Presentation of Malignant Mesothelioma

**DOI:** 10.7759/cureus.43205

**Published:** 2023-08-09

**Authors:** Yusur Alsalihi, Chitra Kandaswamy

**Affiliations:** 1 Medicine, California Health Sciences University, Clovis, USA; 2 Pulmonology, California Health Sciences University, Clovis, USA

**Keywords:** localized malignant pleural mesothelioma, pleura, pleural tumor, lmpm, mediastinal mass, pleural mesothelioma

## Abstract

Localized malignant pleural mesothelioma (LMPM) is a rare cancer with poor survival rates. Often affecting males with asbestos exposure, we report a case of a 56-year-old female with no history of occupational exposure presenting with a worsening cough. A radiological examination revealed left pleural effusion and pleural thickening. Cytological and pathological reports of pleural samples were consistent with malignant mesothelioma of epithelioid type, with the histological examination via video-assisted thoracoscopic surgery (VATS) consistent with a clear cell epithelioid mesothelioma. We discuss the rapid presentation of the disease with emphasis on considering the disease in young patients with no prior asbestos exposure.

## Introduction

Malignant pleural mesothelioma is an aggressive and rare malignancy originating from the mesothelial cells that line the pleural cavity. The median age at diagnosis is 75 years, and overall survival is 38% at one year and 7% at three years, reflecting the poor prognosis [1]. Among females, the diagnosis is even rarer, with the number of deaths due to mesothelioma reported as 614 in 2022 or 4.2 per one million females. The most significant number of deaths was associated with the homemaker occupation (22.8%) [2].

Asbestos exposure is seen as the most significant risk factor for developing mesothelioma. Following reports of prolonged asbestos exposure, an incubation period of 10 years is typically observed in affected patients. During this period, no malignancy is noted. However, there have been rare cases of localized malignant pleural mesothelioma where the malignancy develops within a year [3]. Furthermore, a minority of patients had no documented asbestos exposure, with no definitive risk factor for mesothelioma noted among those cases.

## Case presentation

In March 2022, our 56-year-old female patient began experiencing regular episodes of dry cough. Initially attributing the cough to an asthma exacerbation, she did not seek medical treatment until the following month, presenting to the emergency room of the local hospital complaining of worsening cough and shortness of breath. Patient history was significant for gastroesophageal reflux disease, well-controlled with medication, and Budd-Chiari syndrome. She underwent an ovarian cystectomy at 15 years of age. She is a never-smoker and does not drink. She works in the education system as a principal. Her family history is non-contributory, with both parents alive and healthy. She is married with one son. Subsequent respiratory viral polymerase chain reaction (PCR) panels reported negative findings for COVID-19, respiratory syncytial virus (RSV), parainfluenza, human metapneumovirus, adenovirus, rhinovirus, influenza A, and influenza B. Her basic metabolic panel was normal, and similarly, her complete blood count (CBC) and differential showed normal findings. Her troponin, prothrombin, and international normalized ratio (INR) levels were all normal. Urinalysis showed abnormal findings (Table 1), but no bacteria or WBC were noted in the urine. Lastly, her laboratory results revealed an elevated coccidioides antibodies IgM (cocci Ab IgM) value of 1.12 but an indeterminant antibodies to coccidioides TP antigen (cocci Ab TP).

**Table 1:** Urinalysis laboratory findings

Laboratory value	Result	Normal range
Urine color	Pale yellow	-
Urine clarity	Clear	-
Urine pH	6.3	-
Specific gravity	1.06	-
Glucose	Negative	Negative
Blood	2+	Negative
Protein	Negative	Negative
Leukocyte esterase	1+	Negative
Nitrites	Negative	Negative

She underwent a two-view chest X-ray, which showed a left pleural-based mass and pleural effusion. This was shortly followed by a CT angiogram pulmonary with a contrast scan, which showed a left pleural effusion and 5.5 cm left lobulated pleural-based lung mass in the apex. Findings also showed marked thickening of the pleura in the left upper chest, with no adenopathy or remarkable changes in surrounding bony structures.

The patient was referred to a pulmonologist, and a thoracocentesis was performed, revealing an exudative pleural effusion with a protein level of 4.9 and a lactate dehydrogenase (LDH) level of 173 with 99% predominant lymphocytes (Table 2). This thickening was described as rind-like, with a lobulated border measuring up to 2 cm. The thickening was most prominent in the anterior apical region, spreading laterally along the left superior mediastinal pleura and along the superior aspect of the oblique fissure. No adenopathy was detected, and the bony structures were found unremarkable. The mass showed irregular borders and involved the upper left lung. The present tumor was shaped along the pleural space. Under urgent suspicion of mesothelioma, we referred her to a cardiothoracic surgeon for biopsy and decortication.

**Table 2:** Pleural fluid analysis with findings consistent with exudative pleural effusion NA: not available, LDH: lactate dehydrogenase, WBC: white blood cell, RBC: red blood cell

Laboratory value	Result	Normal reference range
Pleural fluid appearance	Bloody	Clear or pale yellow
Pleural fluid pH	7.1	Negative
Pleural fluid glucose	69 mg/dL	NA
Pleural fluid protein	4 g/dL	NA
Pleural fluid LDH	173 units/L	NA
Pleural fluid WBC count	2,166/uL	≤1,000/uL
Pleural fluid RBC count	42,986/uL	≤0/uL
Pleural fluid protein	4.9 g/dL	NA

**Figure 1:**
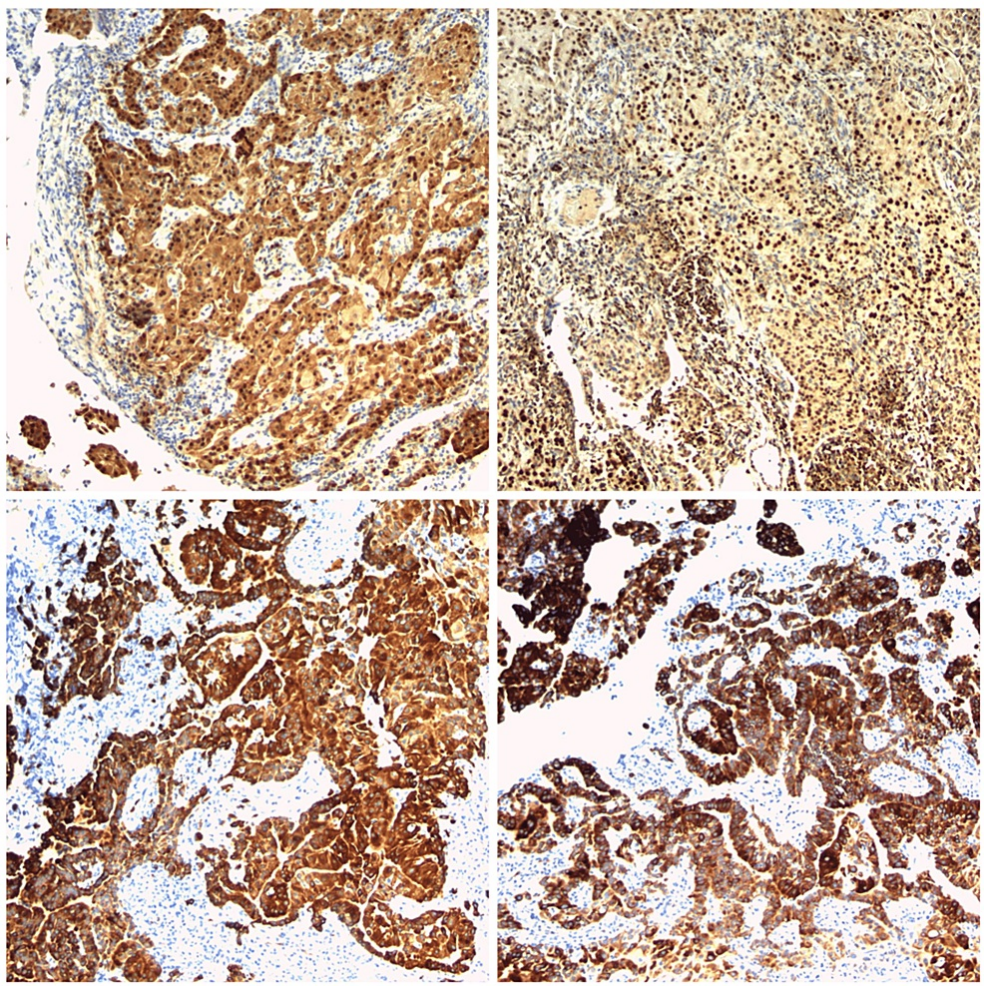
Calretinin staining, GATA3 staining, CK7 staining, and CK5/6 staining (clockwise top left) GATA3: GATA-binding protein 3, CK: cytokeratin

On the following day, she underwent a video-assisted thoracoscopic surgery (VATS) biopsy. Specimens were collected from the left apical parietal pleura and left pleural biopsy. The pathology report showed sheets of epithelioid cells with mild to moderate pleomorphism and prominent nucleoli with scattered large cells. An invasive component was not appreciated since the biopsies only captured the epithelioid cells. Immunohistochemistry stains showed that the epithelioid cells are positive for calretinin, cytokeratin 7 (CK7), CK5/6, D2-40, and Wilms tumor 1 (WT1) (Figure 1). PAX8 staining showed weak, patchy positivity. GATA-binding protein 3 (GATA3) shows variably strong positivity, and the Ki-67 index showed 50% positivity. Immunohistological stains of CK20, thyroid transcription factor 1 (TTF-1), p40, p63, MOC31, B-cell lymphoma 2 (bcl-2), and B72.3 were negative. The report concluded that the immunohistochemical findings are compatible with the malignant mesothelioma epithelioid type. She subsequently underwent a full-body PET/CT scan. No metastatic findings were noted. Furthermore, the patient underwent an echocardiogram, which revealed no pericardial effusion; the inferior vena cava was normal in size, with normal ventricular size. The patient was referred to Stanford to be evaluated for pleurectomy.

She began neoadjuvant therapy with Stanford and underwent three cycles of cisplatin and pemetrexed while evaluating her candidacy for surgery. Her pulmonary function tests on 08/22 revealed a forced vital capacity (FVC) of 2.29 L (65% predicted), forced expiratory volume (FEV1) of 1.76 L (63% predicted), and diffusing capacity of lung for carbon monoxide (DLCO) 42% predicted. These findings are suggestive of mild restriction with moderately reduced DLCO. The second scan showed minimal interval change, with slight improvement regarding the pleural-based disease in the left hemithorax. The plaques appeared thinner by measurement. No lymphadenopathy or other findings to suggest new metastasis was noted. Furthermore, the subcutaneous air in the left lateral chest wall in the prior study was no longer present. The patient is still under consideration for surgery. The patient is pending to have a pleurectomy after the tumor bulk is reduced.

## Discussion

**Figure 2:**
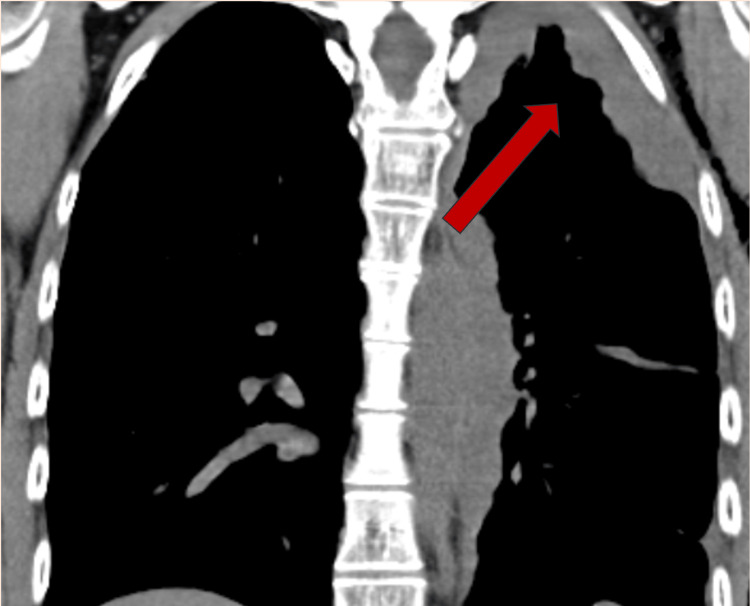
Coronal view with increased left pleural thickening (arrow)

**Figure 3:**
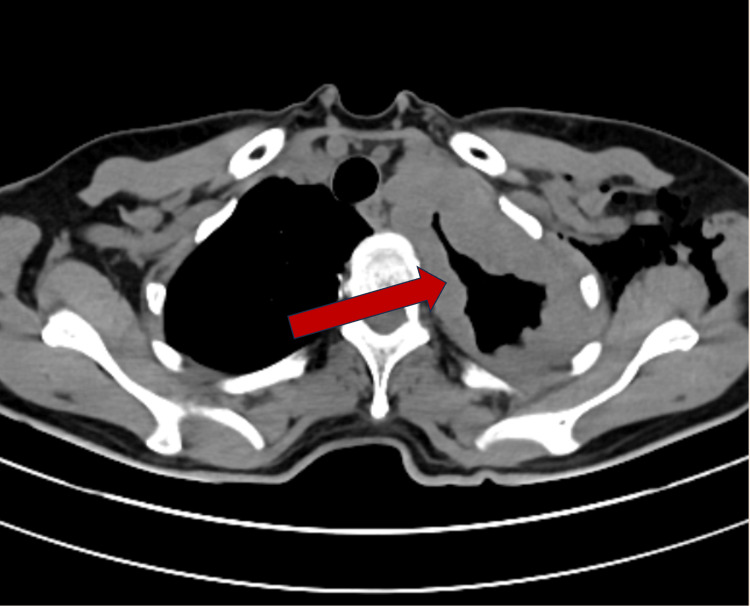
Transverse view showing significant left pleural thickening (arrow)

Our case is that of a 56-year-old female with no prior asbestos exposure, no industrial risk factors, and no symptoms except a worsening cough. No information could be gleaned from the history or clinical presentation to indicate malignant mesothelioma. However, the presence of a mediastinal mass on the left field was the first indication of a plural neoplastic process. The possibility of mesothelioma should be considered in the presence of a mediastinal mass [4]. Mesothelioma was further suspected because of extensive involvement of the mediastinal pleura (Figures 2, 3).

Classification and diagnosis of mesothelial tumors are constantly undergoing modifications and changes. The recent World Health Organization (WHO) classification of mesothelioma in situ as a clinically pathological entity was revised in 2021 and stipulated that diagnosis cannot be based on morphology alone but must demonstrate the loss of biochemical markers BRCA1-associated protein-1 (BAP1) or methylthioadenosine phosphorylase (MTAP) by immunohistochemistry or cyclin-dependent kinase inhibitor 2A (CDKN2A) homozygous deletion [5]. Current guidelines break down mesothelial tumors into benign, pre-invasive, and invasive tumors. The pre-invasive category is further subdivided into adenomatoid tumors, well-differentiated papillary mesothelial tumors, and mesothelioma in situ. Invasive tumors are distinguished as localized or diffuse and are histologically classified into three major subtypes: epithelioid, sarcomatoid, and biphasic [6].

Epithelioid mesothelioma classification by WHO is based on nuclear atypia, mitotic count, and necrosis [6]. A two-tier system of low and high grades is also recommended for treatment management; however, no such system exists for sarcomatoid and biphasic mesotheliomas. Epithelioid tumors are also found to have two architectural patterns: pleomorphic and transitional. Recognizing those cytological features is important to perform the appropriate immunohistochemical workup. The sarcomatoid subtype is characterized by spindle cell proliferation arranged in fascicles or random patterns and is confirmed by immunohistochemistry and ancillary studies, particularly of CDKN2A homozygous deletion or loss of MTAP [6]. This arrangement may invade the adipose tissues and/or lung parenchyma. It has the worst survival rate with an average of four months survival rate in patients who underwent surgical treatment. Furthermore, sarcomatoid mesothelioma has a variant called desmoplastic mesothelioma, which poses a diagnostic challenge due to its histological proximity to pleural hyaline plaque. Lastly, biphasic mesothelioma is composed of both epithelioid and sarcomatoid components. Guidelines stipulate a minimum of 10% of each component for diagnosis.

Treatment options for malignant mesothelioma are limited and challenging. Treatment is based on staging, histological subtype, and the patient’s functional status [7]. In cases of inoperable disease, the patient is assessed for systemic treatment or active symptom control. Another type of management is pleural fluid management, which includes temporary catheterization of the pleural space to drain off fluid [7]. This method also includes talc administration as a therapeutic measure. Surgical options have not been shown to offer a greater survival rate and are often considered for palliative care. The most radical form of surgical treatment is extrapleural pneumonectomy, which offers a modest 18-month survival rate and an overall five-year survival rate of 14%. A recent randomized trial study from the United Kingdom directly compared surgery versus no surgery and showed an overall shorter median survival rate in the surgical group and higher morbidity. Unfortunately, radiotherapy has also shown non-promising results with no overall improvement in survival rates in randomized controlled studies [7].

Determining the subtype and classification is important in determining the prognosis and treatment options and management. This case is clinically significant because it highlights how malignant mesothelioma can present in healthy, young patients with no prior history of asbestos exposure. Clinicians who keep malignant mesothelioma in their differential list while evaluating a mediastinal mass can catch this cancer early, improving survival chances. Currently, the patient is doing well and is undergoing treatment.

## Conclusions

In conclusion, this scientific paper presented a rare case of malignant mesothelioma with an unusual presentation in a 56-year-old female with no history of asbestos exposure. The report emphasized the importance of considering mesothelioma in young patients with no occupational risk factors when evaluating a mediastinal mass. The study highlighted the challenges in diagnosing and managing this aggressive cancer, which has poor survival rates. By understanding the different subtypes and classifications, clinicians can better determine prognosis and treatment options, potentially improving patient outcomes. This case serves as a reminder for healthcare professionals to remain vigilant and consider mesothelioma as a possibility, even in atypical patient profiles.
